# Vitamin B12-Induced Autophagy Alleviates High Glucose-Mediated Apoptosis of Islet β Cells

**DOI:** 10.3390/ijms242015217

**Published:** 2023-10-16

**Authors:** Yu Zhang, Ling Chu, Xi’an Zhou, Tingxia Xu, Qingwu Shen, Tao Li, Yanyang Wu

**Affiliations:** 1 Key Laboratory for Food Science and Biotechnology of Hunan Province, College of Food Science and Technology, Hunan Agricultural University, Changsha 410128, China; zhangyu0918@stu.hunau.edu.cn (Y.Z.); chu13257326523@163.com (L.C.); zhouxian@stu.hunau.edu.cn (X.Z.); 1622143876xtx@stu.hunau.edu.cn (T.X.); yaoyao3153@aliyun.com (Q.S.); 2 Hunan Agricultural Product Processing Institute, Hunan Academy of Agricultural Sciences, Changsha 410125, China; 3 Horticulture and Landscape College, Hunan Agricultural University, Changsha 410128, China; 4 Hunan Co-Innovation Center for Utilization of Botanical Functional Ingredients, Changsha 410128, China; 5 State Key Laboratory of Subhealth Intervention Technology, Changsha 410128, China

**Keywords:** apoptosis, autophagy, diabetes, high glucose, vitamin B12

## Abstract

High glucose levels can lead to the apoptosis of islet β cells, while autophagy can provide cytoprotection and promote autophagic cell death. Vitamin B12, a water-soluble B vitamin, has been shown to regulate insulin secretion and increase insulin sensitivity. However, the precise mechanism of action remains unclear. In this study, we investigated the influence of vitamin B12 on high glucose-induced apoptosis and autophagy in RIN-m5F cells to elucidate how vitamin B12 modulates insulin release. Our results demonstrate that exposure to 45 mM glucose led to a significant increase in the apoptosis rate of RIN-m5F cells. The treatment with vitamin B12 reduced the apoptosis rate and increased the number of autophagosomes. Moreover, vitamin B12 increased the ratio of microtubule-associated protein 1 light chain 3 beta to microtubule-associated protein 1 light chain 3 alpha (LC3-II/LC3-I), while decreasing the amount of sequestosome 1 (p62) and inhibiting the phosphorylation of p70 ribosomal protein S6 kinase (p70S6K) under both normal- and high-glucose conditions. The additional experiments revealed that vitamin B12 inhibited high glucose-induced apoptosis. Notably, this protective effect was attenuated when the autophagy inhibitor 3-methyladenine was introduced. Our findings suggest that vitamin B12 protects islet β cells against apoptosis induced by high glucose levels, possibly by inducing autophagy.

## 1. Introduction

Vitamin B12, also known as cobalamin, is a water-soluble B-complex vitamin containing a central cobalt ion coordinated by a corrin ring. Vitamin B12 has the highest molecular weight and most complex structure of all the vitamins and is the only vitamin that contains a metal ion. Vitamin B12 comes in numerous forms [1]. In particular, methylcobalamin is one of the active forms used in mammalian cell metabolism, while cyanocobalamin is the main form found in the diet and is commonly known as dietary vitamin B12 [2]. Vitamin B12 is an important micronutrient for one-carbon metabolic pathways. As a coenzyme of methionine synthase, it is an indispensable key compound for the synthesis of methionine and the crucial methyl donor S-adenosylmethionine (SAM) derived from it. Moreover, vitamin B12 plays an important role in the process of DNA methylation. When vitamin B12 is deficient or metabolized abnormally, it may hinder the formation of methyl compounds, thus leading to genomic instability events such as DNA methylation and abnormal gene expression and even increasing the risk of developmental abnormalities and degenerative diseases of the nervous system as well as cardiovascular diseases. Vitamin B12 is synthesized by specific bacteria (such as intestinal bacteria) and cannot be synthesized by plants and mammals themselves. Therefore, mammals must obtain vitamin B12 through their dietary intake.

Some studies have found that diabetic individuals who take metformin for a prolonged period are susceptible to vitamin B12 deficiency [3]. During cellular metabolism, vitamin B12 is gradually consumed by the methionine cycle, and vitamin B12 deficiency appears to be associated with an increased risk of diabetes [4]. In a multicenter randomized double-blind controlled trial, it was found that high-dose vitamin B supplementation (folic acid, vitamin B6, and vitamin B12) significantly reduced the plasma total homocysteine level in patients with diabetic nephropathy [5]. It is preferable to supplement two or more B-complex vitamins at the same time. The risk factors for the occurrence and development of diabetes also include oxidative stress and advanced glycation end products of proteins [6]. Folic acid, vitamin B6, and vitamin B12 have anti-saccharification effects and can help improve islet β cell function by reducing oxidative stress [7].

Diabetes is a metabolic disease characterized by hyperglycemia. Long-term hyperglycemia can lead to the chronic damage and dysfunction of various tissues, especially the eyes, kidneys, cardiovascular system, and nerves [8,9]. According to its etiology, diabetes can be divided into four categories: type I diabetes mellitus (T1DM), type II diabetes mellitus (T2DM), other special diabetes, and diabetes during pregnancy. T1DM is primarily caused by the destruction of pancreatic islet β cells due to factors such as autoimmunity, which leads to insulin deficiency in the body. T2DM is often accompanied by insulin secretion disorders of varying degrees due to insulin resistance, which can be caused by various factors. Other special diabetes may result from insulin deficiency, pancreatic exocrine diseases, islet β cell function gene defects, chemical exposure, or drug infection [10]. Gestational diabetes, which typically occurs in middle or late pregnancy, is mostly related to insulin resistance [11]. Numerous clinical studies and epidemiological evidence indicate that vitamin B12 may be involved in the pathogenesis of glucose intolerance. It can reduce insulin resistance and oxidative stress by regulating the synthesis of homocysteine, thus decreasing the risk of diabetes [12,13,14,15,16].

Autophagy is a membrane transport mechanism that delivers cytoplasmic proteins and dysfunctional organelles to lysosomes for degradation to achieve cell renewal [17]. Autophagy usually occurs primarily to maintain the protein balance in the body and cellular function during organelle turnover. The stress of pathological processes, such as hypoxia, loss of growth factors, starvation, or excessive release of reactive oxygen species, increases the cellular demand for nutrition and energy [18]. At this time, some components of cells, such as proteins and redundant or damaged organelles, are degraded and recycled through autophagy to ensure cell stability.

T2DM is a metabolic disease characterized by chronic hyperglycemia. Its basic pathology is insulin resistance or islet β cell dysfunction, which leads to the relative or absolute insufficiency of insulin secretion and in turn causes metabolic abnormalities relating to energy, carbohydrates, fats, and proteins [19]. Autophagy is an important mechanism regulating cell growth and development, energy metabolism, immune response, and other processes, as well as a unique self-protection mechanism of eukaryotic cells that is often associated with apoptosis [20]. Increased apoptosis is considered the key mechanism underlying the reduction in and dysfunction of pancreatic islet β cells in T2DM [21]. Cell autophagy is a kind of self-stabilization mechanism that widely exists in eukaryotic cells, and the dysfunction of this process has been implicated in the occurrence of many human diseases [22]. Following key breakthroughs in genetic studies involving various fungi, especially Saccharomyces cerevisiae, research into the basic mechanism of autophagy and its physiological relationship with health and disease has seen explosive growth [23]. However, the interaction between autophagy and apoptosis is very complex, and comprehensive and in-depth research into this topic is still needed to reveal strategies for the improvement of islet β cell dysfunction and the treatment of T2DM.

Recently, dietary intervention and exercise have been proposed as effective approaches for alleviating the T2DM. Here we report that vitamin B12 induces autophagy via the mammalian target of rapamycin (mTOR)-dependent pathway. Further experiments confirmed that vitamin B12-induced autophagy alleviated high glucose-induced islet β cell apoptosis. Our results are expected to provide useful insights for the application of foods rich in vitamin B12 to alleviate T2DM.

## 2. Results

### 2.1. Vitamin B12 Inhibits High Glucose-Induced Apoptosis

**Figure 1:**
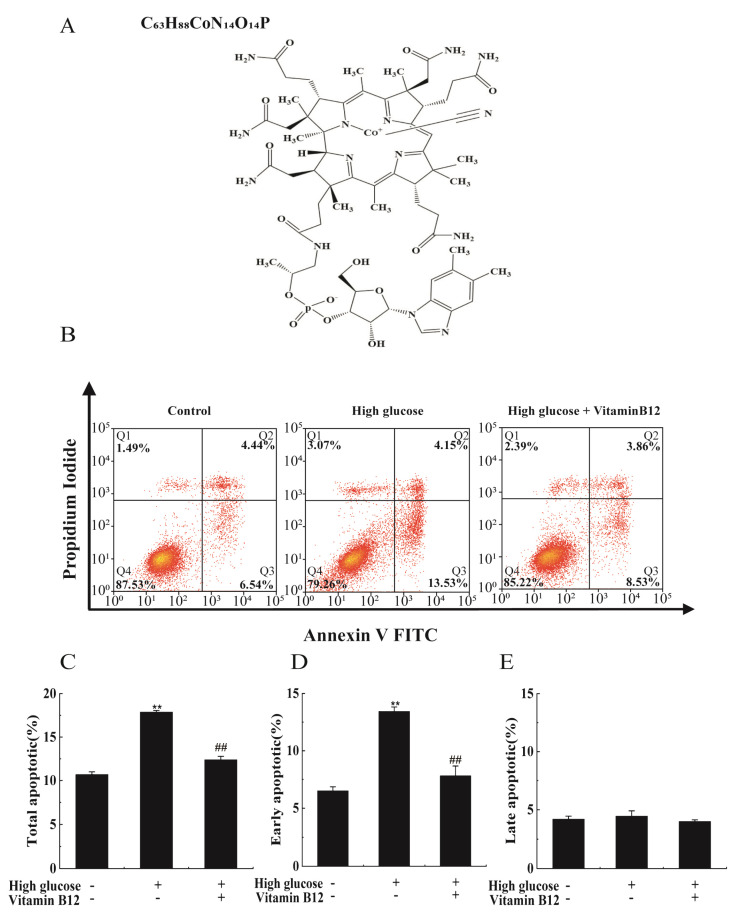
Vitamin B12 exhibited a protective effect against high glucose-induced apoptosis in cells. (**A**) The structure diagram of vitamin B12. (**B**) The apoptosis rates of RIN-m5F cells were evaluated using flow cytometry after 36 h of culture with 45 mM glucose or with 2 μM vitamin B12 plus 45 mM glucose. (**C**–**E**) The total apoptosis, late apoptosis, and early apoptosis were analyzed in cells subjected to the treatments described in (**B**). All experiments were conducted a minimum of three times. The error bars represent the standard deviation, and statistical analysis using one-way ANOVA revealed significant differences. ** *p* < 0.01 compared to the control group, ## *p* < 0.01 compared to the high glucose-treated group.

To explore the effect of vitamin B12 on apoptosis induced by high glucose concentrations, we exposed RIN-m5F cells to high glucose stress while simultaneously administering vitamin B12. Flow cytometry was then utilized to assess the rate of apoptosis. The results indicated that the presence of 45 mM glucose resulted in a noteworthy increase in the apoptosis rate, elevating it from 10.98% to 17.68%. However, the addition of vitamin B12 effectively mitigated apoptosis, reducing the rate from 17.68% to 12.39% (Figure 1).

### 2.2. Vitamin B12 Induces Autophagy under Normal Culture Conditions

**Figure 2:**
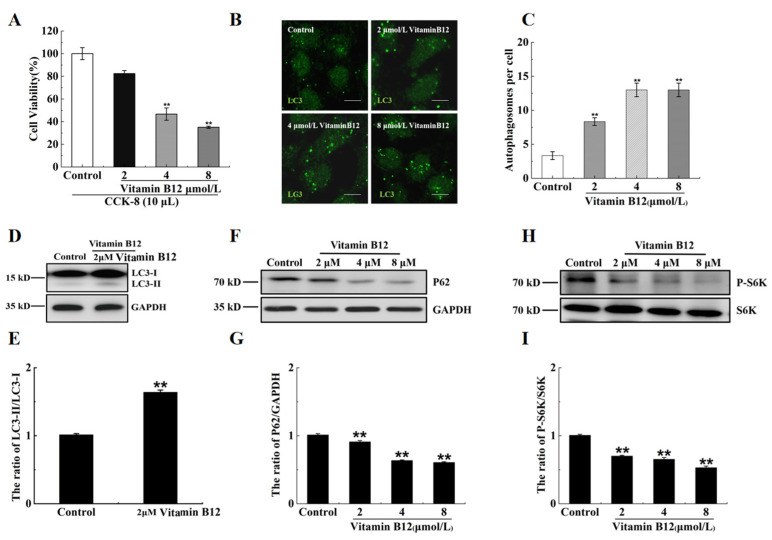
Vitamin B12 induced autophagy in RIN-m5F cells. (**A**) RIN-m5F cells were subjected to varying concentrations of vitamin B12, and cytotoxicity levels were assessed utilizing a CCK-8 kit assay. (**B**) The cells were treated with vitamin B12 concentrations of 2, 4, and 8 μM for 24 h and stained utilizing an anti-LC3 antibody (scale = 5 μm). (**C**) The cells were cultured as described in section (**B**), and the mathematical statistical methods were conducted to calculate the number of autophagosomes in each cell. At least 30 cells were counted for statistical analysis utilizing the *t*-test. (**D**,**F**,**H**) The impact of vitamin B12 at 2, 4, and 8 μM concentrations on the expression of autophagy-related proteins (anti-LC3, anti-p62, anti-GAPDH, anti-S6K, and an-ti-p-S6K) in cells were carried out using Western blotting. (**E**,**G**,**I**) The LC3-II/LC3-I, p62/GAPDH, and p-S6K/S6K ratios in (**D**,**F**,**H**), respectively, were analyzed by integrated optical density (IOD) using Image-Pro Plus 6.0 software. All experiments were conducted a minimum of three times. The error bars represent the standard deviation, ** *p* < 0.01 reveals significant differences in comparison to the control group.

It has been reported that the total stores of vitamin 12 in the human body are between 1.1 and 3.9 mg, with a loss of 0.13% per day. The content of vitamin B12 in serum is between 96 and 148 pmol/L [24]. In this work, the islet β cells were treated with 2, 4, and 8 μM vitamin B12, and the cell survival rates were determined by performing cell counting kit-8 (CCK-8) assays. The survival rate of the cells treated with 2 μM vitamin B12 did not exhibit a significant difference compared with the control group, whereas the cells treated with 4 or 8 μM vitamin B12 displayed lower survival rates (Figure 2A). This suggests that after the addition of 2 μM vitamin B12, cytoplasmic microtubule-associated protein 1 light chain 3 alpha (LC3-I) is converted to microtubule-associated protein 1 light chain 3 beta (LC3-II) and binds to the autophagic membrane. The number of LC3-positive puncta and the LC3-II/LC3-I ratio were utilized to assess the degree of autophagy. To examine the impacts of vitamin B12 on autophagy, the cells were subjected to vitamin B12 treatment, and the quantification of the autophagosomes was conducted through immunofluorescence staining. The results revealed an increase in the number of autophagosomes in the cells treated with 2 μM vitamin B12 (Figure 2B,C). In addition, the vitamin B12-treated cells exhibited a significantly higher LC3-II/LC3-I ratio than the control group (Figure 2D,E).

Sequestosome 1 (p62) is a substrate protein that specifically undergoes degradation during autophagy. Vitamin B12 markedly reduced the protein expression of p62, illustrating its involvement in autophagy (Figure 2F,G). As mentioned by Lu et al. [25], mTOR acts as a negative regulator of autophagy. The phosphorylation level of the substrate protein p70 ribosomal protein S6 kinase (p70S6K) serves as an indicator of mTOR activity, Where a decrease in p70S6K phosphorylation indicates mTOR inhibition, as reported by Shin et al. [26]. Our data revealed that vitamin B12 inhibited the phosphorylation of p70S6K (Figure 2H,I). On the basis of these results, it was concluded that vitamin B12 possesses the capability to induce autophagy under normal culture conditions.

### 2.3. Vitamin B12 Induces Autophagy under High Glucose Stress

**Figure 3:**
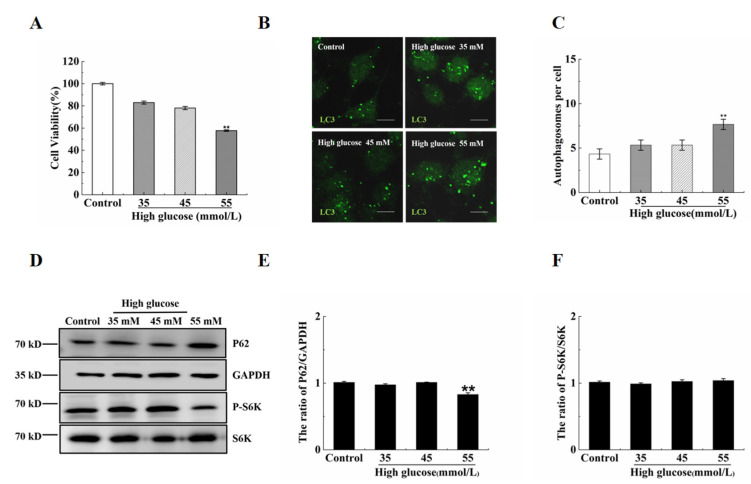
The influence of high glucose on autophagy in RIN-m5F cells. (**A**) RIN-m5F cells were exposed to the indicated concentrations of glucose, and cytotoxicity was assessed utilizing a CCK-8 kit assay. (**B**) The cells were cultured under conditions of 35, 45, and 55 mM glucose for 36 h and then stained with anti-LC3 antibody (scale = 5 μm). (**C**) The cells were treated according to section (**A**), mathematical statistical methods were conducted to calculate the number of autophagosomes per cell, and at least 30 cells were counted by statistical analysis based on a *t*-test. (**D**) The cells were treated according section (**A**), and Western blotting analysis was conducted to investigate the expression levels of autophagy-related proteins with specific antibodies including anti-LC3, anti-p62, anti-GAPDH, anti-S6K, and anti-p-S6K. (**E**,**F**) Subsequent to the procedure described in section (**D**), the ratios of p62/GAPDH and p-S6K/S6K were assessed through IOD measurements utilizing Image-Pro Plus 6.0 software. All experiments were conducted a minimum of three times. The error bars represent the standard deviation, ** *p* < 0.01 reveals significant differences in comparison to the control group.

Hyperglycemia is a common clinical feature of diabetes. In this study, we aimed to investigate the influence of vitamin B12 on autophagy under conditions of high glucose stress. To assess this, RIN-m5F cells were exposed to various concentrations of glucose (35, 45, and 55 mM) (Figure 3A). We then examined the number of autophagosomes and the LC3-II/LC3-I ratio as indicators of autophagy. Surprisingly, our results revealed no significant change in the number of autophagosomes in the cells treated with 35 or 45 mM high glucose compared with the control group (Figure 3B,C). In addition, the p62 expression and p70S6K phosphorylation level remained unaltered in the cells treated with 35 or 45 mM glucose, whereas they significantly decreased in the cells treated with 55 mM glucose (Figure 3D,E). Furthermore, we assessed the lysosome degradation activity and found that treatment with vitamin B12 or 45 mM glucose plus vitamin B12 did not impair lysosome function (Figure 3F). Collectively, these findings indicate that vitamin B12 can induce autophagy under normal- or high-glucose stress conditions.

**Figure 4:**
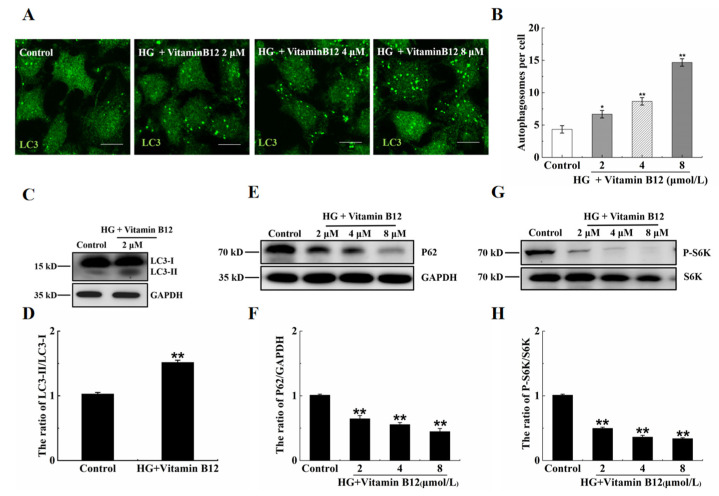
Vitamin B12 induced autophagy under conditions of high glucose stress. (**A**) RIN-m5F cells were treated with varying concentrations of vitamin B12 (2, 4, and 8 μM) in conjunction with 45 mM glucose for 36 h, and the number of autophagosomes was determined through immunofluorescence analysis (scale = 5 μm). (**B**) The cells were treated according to section (**A**), and mathematical statistical methods were conducted to calculate the number of autophagosomes in each cell. At least 30 cells were counted by t test analysis. (**C**,**E**,**G**) The cells were treated according to section (**A**), and Western blotting was conducted to investigate the expression levels of proteins with specific anti-LC3, anti-p62, anti-GAPDH, anti-S6K, and anti-p-S6K antibodies. (**D**,**F**,**H**) The ratio of LC3-II/LC3-I, p62/GAPDH and p-S6K/S6K ratios in (**C**,**E**,**G**), respectively, were assessed through IOD measurements utilizing Image-Pro Plus 6.0 software. All experiments were conducted a minimum of three times. The error bars represent the standard deviation, ** *p* < 0.01, * *p* < 0.05 reveals significant differences in comparison to the control group.

To further elucidate the effects of vitamin B12 on autophagy under high glucose stress, the cells were exposed to vitamin B12 (2 μM) under high-glucose conditions for 36 h. The number of autophagosomes increased in a dose-dependent manner with the addition of vitamin B12 (Figure 4A,B). Moreover, the ratio of LC3-II/LC3-I and degradation rate of p62 increased, while the phosphorylation of p70S6K decreased at higher concentrations of vitamin B12 (Figure 4C–H). These data indicate that vitamin B12-induced autophagy may be mediated by the inhibition of mTOR phosphorylation under conditions of high glucose stress. Overall, the obtained results demonstrate that vitamin B12 can effectively induce autophagy under conditions of high glucose stress.

### 2.4. 3-Methyladenine Suppresses Vitamin B12-Induced Autophagy under High Glucose Stress

**Figure 5:**
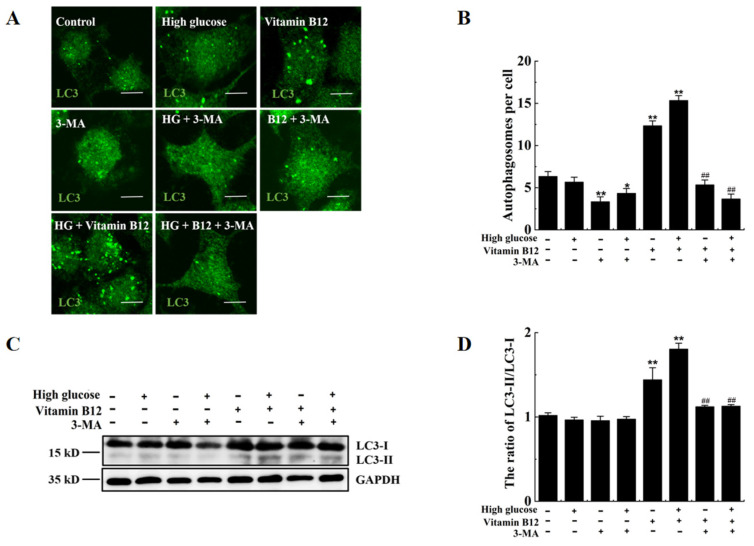
3-MA suppressed vitamin B12-induced autophagy under the condition of high glucose stress. (**A**) RIN-m5F cells were cultivated under various conditions: either in the presence or absence of 45 mM glucose, supplemented with 10 mM 3-MA, a combination of 45 mM glucose and 10 mM 3-MA, 2 μM vitamin B12, 45 mM glucose plus 2 μM vitamin B12, or a concoction of 20 mM glucose, 2 μM vitamin B12, and 10 mM 3-MA, cultured for 36 h and stained with anti-LC3 anti-body (scale bar = 5 μm). (**B**) The cells were treated according to section (**A**), and mathematical statistical methods were conducted to calculate the number of autophagosomes in each cell. At least 30 cells were analyzed. (**C**) The cells were treated according to section (**A**), and Western blotting was performed to investigate the expression levels of proteins with specific antibodies against LC3 and GAPDH. (**D**) The LC3-II/LC3-I ratio in (**C**) was analyzed by IOD analysis utilizing Image-Pro Plus 6.0 software. All experiments were conducted a minimum of three times. The error bars represent the standard deviation, ** *p* < 0.01, * *p* < 0.05 reveals significant differences in comparison to the control group. ## *p* < 0.01 reveals significant differences in comparison to the group treated with vitamin B12 combined with high glucose through one-way ANOVA.

To investigate the effects of 3-methyladenine (3-MA), an autophagy inhibitor, on vitamin B12-induced autophagy under conditions of high glucose stress, the number of autophagosomes and the LC3-II/LC3-I ratio in the β-islet cells were examined. The results revealed significant increases in the number of autophagosomes and the LC3-II/LC3-I ratio in the cells treated with vitamin B12 and glucose. However, these increases were reversed upon the addition of 3-MA (Figure 5). Thus, it was concluded that 3-MA inhibits vitamin B12-induced autophagy under conditions of high glucose stress.

### 2.5. 3-MA Inhibits Vitamin B12-Induced Cytoprotective Autophagy against Apoptosis

**Figure 6:**
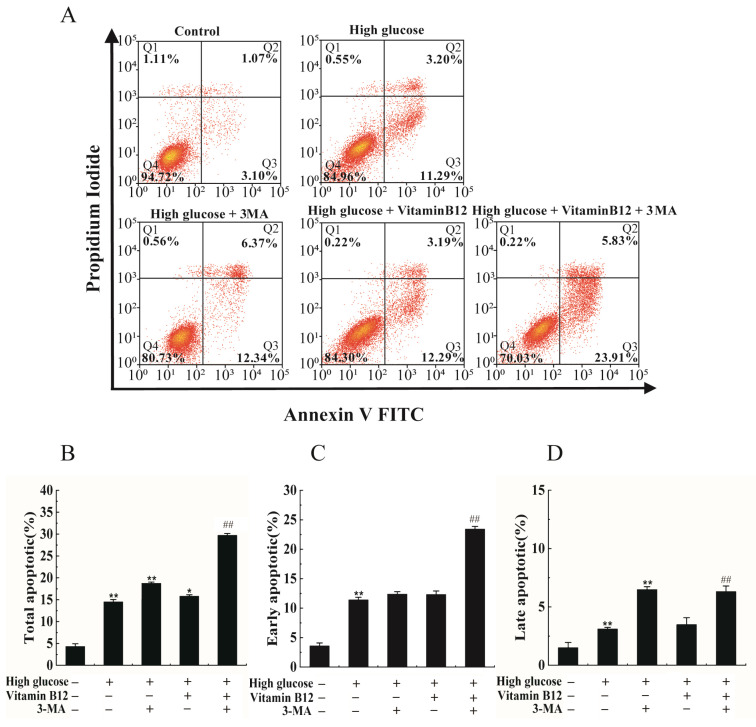
Vitamin B12 exhibited a protective effect on RIN-m5F cells from apoptosis by promoting autophagy under the condition of high glucose stress. (**A**) RIN-m5F cells were cultured with various conditions: either in the absence or presence of 45 mM glucose, or combined with additional agents such as 10 mM 3-MA, 2 μM vitamin B12, or a concoction of 10 mM 3-MA and 2 μM vitamin B12, and the rate of apoptosis was evaluated using flow cytometry techniques. (**B**–**D**) The cells were treated according to section (**A**), and total apoptosis, late apoptosis, and early apoptosis rates were analyzed. All experiments were conducted a minimum of three times. The error bars represent the standard deviation, ** *p* < 0.01, * *p* < 0.05 reveals significant differences in comparison to the control group. ## *p* < 0.01 reveals significant differences in comparison to the group treated with vitamin B12 combined with high glucose through one-way ANOVA.

To explore the potential mechanism of vitamin B12 against apoptosis induced by high glucose concentrations, experiments were conducted in which the cells were subjected to high glucose stress and treated with a combination of vitamin B12 and 3-MA. The apoptosis rate was subsequently assessed by flow cytometry. The addition of vitamin B12 was found to reduce the total apoptosis rate of the islet β cells from 18.71% to 14.49% (Figure 6). However, when 3-MA was added, the total apoptosis rate markedly increased from 14.49% to 29.74%. These findings strongly suggest that vitamin B12 exerts an inhibitory effect on high glucose-induced apoptosis in RIN-m5F cells, potentially through autophagy pathways.

## 3. Discussion

T2DM is primarily attributable to insulin resistance and insulin secretion disorders caused by various factors, often leading to severe complications such as diabetic retinopathy, diabetic nephropathy, skin infections, and osteoporosis [27,28]. T1DM is also caused by multiple factors that induce islet β cell destruction [29]. The pathogenesis is complex for both T2DM and T1DM, and both are influenced by genetic factors, environmental factors, lifestyle choices, meal timing, physical activity, and diet [30,31,32,33,34]. The risk of diabetes is also affected by age, gender, and race, with age in particular being a primary risk factor [35]. Men have a higher prevalence of diabetes than women, and high levels of insulin receptor substrate 4 and serum total cholesterol as well as elevated blood pressure are also associated with a higher risk of diabetes [10,36,37]. Unhealthy lifestyle choices, such as smoking, excessive alcohol consumption, high-fat diets, and a lack of exercise, also play a role. High-fat diets often lead to poor microbiome health, which in turn disrupts the balance of gut bacteria and results in dysbiosis. This dysbiosis can contribute to metabolic dysfunction, such as elevated insulin resistance and inflammation, both of which play significant roles in the development of T2DM [38]. Studies have indicated a correlation between diet and diabetes, with the increased consumption of yogurt, vegetables, fiber, and fruits and the decreased consumption of processed meats and seafood reducing the risk of diabetes [39]. Numerous epidemiological studies and clinical trials suggest that vitamin B12 may play a role in the pathogenesis of glucose intolerance, reducing insulin resistance and oxidative stress by regulating homocysteine synthesis and thus decreasing diabetes risk [3,7,40].

Autophagy is a cellular protein and organelle recycling mechanism that is critical to the growth, development, and aging of eukaryotic cells [41]. This process is regulated by autophagy-related genes (ATG), with ATG1 to ATG10, ATG12 to ATG14, ATG16 to ATG18, ATG29, and ATG31 being essential [42], and also by various signal transduction pathways, with mTOR being a key negative regulatory kinase, and the type I phosphatidylinositol 3 kinase/protein kinase B (PI3K/AKT) signal pathway and extracellular signal activated kinase 1/2 (ERK1/2) signal pathway activating mTOR to inhibit autophagy. Conversely, the AMP-activated protein kinase (AMPK) pathway and p53 signal transduction pathway inhibit mTOR to activate autophagy [43,44].

Notably, autophagy levels in pancreatic β-cells vary in different injury environments, influencing pancreatic β-cell function, with autophagy protecting pancreatic β-cell function and inducing apoptosis in some cases [44]. Basic autophagy regulates the normal metabolism of pancreatic β-cells, with ATG7 being an essential autophagy effector enzyme. Deficient autophagy in mice resulting from the knockout of the autophagy-related gene Atg7 leads to impaired glucose tolerance, reduced insulin levels, decreased pancreatic β-cell count, and hyperglycemia [45,46].

Metformin is the most commonly prescribed oral hypoglycemic drug for T2DM treatment [47]. Vitamin B12 intake plays an essential role in single-carbon metabolism. A large prospective randomized trial demonstrated that dietary supplementation with folic acid, vitamin B6, and vitamin B12 for five years reduced the homocysteine levels and overall stroke risk by 25% in high-risk patients, including those with pre-existing diabetes and other risk factors [7,48]. Vitamin B12 may provide a novel function in preventing diabetes and related complications, particularly in individuals taking metformin at doses exceeding 2000 mg/day and lasting over four years [3,49]. Hyperglycemia activates MAPK/ERK signaling, induces the formation of misfolded and abnormal proteins, and leads to cell apoptosis [50]. Our data also revealed that vitamin B12 decreased the rate of high glucose-induced apoptosis from 17.68% to 12.39%. Consequently, there exists the possibility that vitamin B12 inhibits apoptosis by correcting the misfolding of proteins, which will be one subject of our future research.

In summary, the relationship between vitamin B12 intake and diabetes risk remains controversial. Further prospective cohort studies and experimental research are necessary to explore the relationship between vitamin B intake and diabetes. Reasonable changes in dietary habits and lifestyle choices can provide effective guidance and help in preventing diabetes, reducing the incidence of the complications, disability, and mortality rates associated with diabetes and ultimately improving patients’ quality of life. It has also been reported that high glucose concentrations induce osmotic stress and lead to apoptosis [51]. Furthermore, the role of autophagy on osmotic-induced apoptosis needs to be explored.

## 4. Materials and Methods

### 4.1. Reagents and Antibodies

Vitamin B12 (8040) was obtained from Beijing Solarbio Biological Technology Co., Ltd. (Shanghai, China). The following antibodies were purchased from various sources: anti-LC3 polyclonal antibody (PM036), anti-LC3 monoclonal antibody (M186-3), and anti-p62 antibody (PM045) from Medical Biological Laboratory; anti-p70S6K antibody (2708) and anti-phosphorylated p70S6K antibody (9206) from Cell Signaling Technology (Beverly, MA, USA); and anti-3-phosphoglyceraldehyde dehydrogenase (GAPDH) anti-body (ZB002) from YTHX Biotechnology Co., Ltd. (Beijing, China). For flow cytometry analysis, propidium iodide (PI) and annexin V fluorescein isothiocyanate (FITC) were acquired from BD Biotechnology Research Co., Ltd. (San Jose, CA, USA), while goat anti-mouse IgG (1070-05) and goat anti-rabbit (4050-05) antibodies were purchased from Southern Biotechnology Company (Birmingham, UK). Finally, Alexa Fluor 488 goat anti-rabbit antibody (A11034) was obtained from Invitrogen Life Technology (Shanghai, China).

### 4.2. Cell Culture

RIN-m5F cells were cultured according to standard conditions at 37 °C under 5% CO_2_ atmosphere. The culture medium used was standard RPMI 1640 containing 4500 mg/L glucose supplemented with 10% fetal bovine serum (FBS) obtained from Biological Industries, Israel.

### 4.3. Immunofluorescence Staining

RIN-m5F cells were cultured in 24-well plates with round cover glasses. The cells were subjected to a series of steps for preparation and imaging. First, the cells were washed three times with phosphate-buffered saline (PBS) and then fixed with 4% paraformaldehyde for 10 min. After fixation, the cells were washed again with PBS and sealed using PBS containing 10% FBS for 30 min. Next, the cells were incubated with anti-LC3 antibody at 37 °C for 1 h then washed again with PBS. Subsequently, the cells were incubated with Alexa Fluor 488 goat anti-rabbit antibody at 37 °C for 1 h. Finally, the cells were sealed using a fluorescence quencher and examined via a confocal microscope (Zeiss LSM 710, Jena, Germany).

### 4.4. Western Blotting

RIN-m5F cells were cultured in 6-well plates. The growth medium was then discarded and the cells were rinsed three times with PBS. A solution of 2% sodium dodecyl sulfate (SDS) was applied to the cells and the resulting cellular extract was subjected to heat treatment at 100 °C for 10 min then combined with 6× protein loading buffer (J21020, Transgen). The proteins present in the extract were then separated by SDS-PAGE and electrophoretically transferred to nitrocellulose membranes. Next, the membranes were blocked with a solution of 5% skim milk powder for 1 h, and then incubated overnight at 4 °C with the appropriate primary antibodies. After three washes with PBST, the membranes were incubated with the appropriate secondary antibodies for 1 h. Finally, the membranes were imaged using an Image Quant LAS4000 Mini system (GE Healthcare Bio-Sciences AB, Uppsala, Sweden) after brief incubation with Western-Bright ECL chemiluminescent HRP substrate (32106, SuperSignal West Dura, Thermo Pierce, Waltham, MA, USA).

### 4.5. Cell Apoptosis Assay

To analyze apoptotic cells, we utilized the PI-Annexin V-FITC Apoptosis Detection Kit in conjunction with flow cytometry. RIN-m5F cells were cultured in 12-well plates for either 24 or 36 h then washed with PBS and digested with trypsin. The cells were collected by centrifugation and resuspended in 100 μL of binding buffer. Each tube containing the cell suspension was then stained with 5 μL of annexin V-FITC and 5 μL of PI for 15 min. To complete the staining process, 400 μL of binding buffer was added. Subsequently, the fluorescence intensities of the stained cells were analyzed using a flow cytometer (Beckman MoFlo XDP, Georgia, GA, USA).

### 4.6. Cell Viability Assay

The cells were seeded in 96-well plates at a density of 105 cells/well and incubated for 24 h. Next, the appropriate substances were added to each well, and the plates were incubated at 37 °C for a specific duration. Afterward, 10 μL of CCK8 solution was introduced into each well, and the plates were further incubated at 37 °C for 1–4 h. The optical density at 450 nm was determined using a microplate reader.

## 5. Conclusions

In this work, we found that vitamin B12 reduces high glucose-induced apoptosis in RIN-m5F cells. The results indicated that vitamin B12 addition increases the number of autophagosomes and the LC3-II/LC3-I ratio, while decreasing p62 expression and p70S6K phosphorylation. Vitamin B12 inhibited high glucose-induced apoptosis and this protection was attenuated by the autophagy inhibitor 3-MA. These findings indicate that vitamin B12 may protect islet β cells through the induction of autophagy via the mTOR signaling pathway.

## Data Availability

The data used to support the findings of this study are included within the article.
